# Evaluation of the inflammatory markers CCL8, CXCL5, and LIF in patients with anastomotic leakage after colorectal cancer surgery

**DOI:** 10.1007/s00384-020-03582-2

**Published:** 2020-04-19

**Authors:** F. Klupp, S. Schuler, C. Kahlert, N. Halama, C. Franz, P. Mayer, T. Schmidt, A. Ulrich

**Affiliations:** 1 Department of General, Visceral and Transplantation Surgery, University of Heidelberg, Im Neuenheimer Feld 110, 69120 Heidelberg, Germany; 2 Department of Visceral, Thoracic and Vascular Surgery, University of Dresden, Fetscherstr. 74, 01307 Dresden, Germany; 3 National Center for Tumor Diseases, Medical Oncology and Internal Medicine VI, Tissue Imaging and Analysis Center, Bioquant, University of Heidelberg, Im Neuenheimer Feld 267, 69120 Heidelberg, Germany; 4 Department of Diagnostic and Interventional Radiology, Heidelberg University Hospital, Im Neuenheimer Feld 110, 69120 Heidelberg, Germany; 5 Department of General and Visceral Surgery, Lukas Hospital Neuss, Preußenstr. 84, 41464 Neuss, Germany

**Keywords:** Anastomotic leakage, Colorectal cancer, CCL8, CXCL5, LIF

## Abstract

**Purpose:**

Anastomotic leakage constitutes a dreaded complication after colorectal surgery, leading to increased morbidity and mortality as well as prolonged hospitalization. Most leakages become clinically apparent about 8 days after surgery; however, early detection is quintessential to reduce complications and to improve patients’ outcome. We therefore investigated the significance of specific protein expression profiles as putative biomarkers, indicating anastomotic leakage.

**Methods:**

In this single-center prospective cohort study serum and peritoneal fluid samples—from routinely intraoperatively inserted drainages—of colorectal cancer patients were collected 3 days after colorectal resection. Twenty patients without anastomotic leakage and 18 patients with an anastomotic leakage and without other complications were included. Protein expression of seven inflammatory markers in serum and peritoneal fluid was assessed by multiplex ELISA and correlated with patients’ clinical data.

**Results:**

Monocyte chemoattractant protein 2 (CCL8/MCP-2), leukemia-inhibiting factor (LIF), and epithelial-derived neutrophil-activating protein (CXCL5/ENA-78) were significantly elevated in peritoneal fluid but not in serum samples from patients subsequently developing anastomotic leakage after colorectal surgery. No expressional differences could be found between grade B and grade C anastomotic leakages.

**Conclusion:**

Measurement 3 days after surgery revealed altered protein expression patterns of the inflammatory markers CCL8/MCP2, LIF, and CXCL5/ENA-78 in peritoneal fluid from patients developing anastomotic leakage after colorectal surgery. Further studies with a larger patient cohort with inclusion of different variables are needed to evaluate their potential as predictive biomarkers for anastomotic leakage.

**Electronic supplementary material:**

The online version of this article (10.1007/s00384-020-03582-2) contains supplementary material, which is available to authorized users.

## Introduction

Anastomotic leakage (AL) after colorectal surgery constitutes a dreaded complication after colorectal surgery [1]. Depending on the anatomical localization, insufficiency rates of about 3% after colon surgery and between 3 and 19% after colorectal surgery are described [2–4]. Even in high-volume centers, a portion of patients develops an anastomotic leakage. However, high-volume centers reveal less in-hospital mortality rates of patients with anastomotic leakage [5] . Consensus definition states anastomotic leakage as a communication between the intra- and extraluminal compartments resulting from a defect in the integrity of the intestinal wall at the anastomosis. Leakages originating from the suture or staple line of a neorectal reservoir, as well as a pelvic abscess in the proximity of the anastomosis, are also considered an anastomotic leakage [6, 7]. Defined by the International Study Group of Rectal Cancer, three grades of AL exist ranking the AL due to its clinical severity. Grade A is called a radiologic leakage meaning the patient has no clinical symptoms or increased infectious values in the blood test. This kind of anastomotic leakage entails no active therapeutic intervention. Grade B patients present with leukocytosis, an increase of CRP, abdominal pain, and distension and require an active therapeutic intervention in terms of antibiotics or an interventionally placed pelvic drain. But there is no need for relaparotomy. Grade C AL includes the symptoms as Grade B together with an ensuing peritonitis or sepsis. Patients with Grade C AL require a relaparotomy which is often associated with Hartmann’s procedure [6, 7]. The mean occurrence of colorectal anastomotic leakage (CAL) has been described for the eighth postoperative day (POD), with an interval between the sixth and thirteenth POD [8, 9]. Preoperative and intraoperative risk factors for CAL are male sex, distal anastomosis, advanced tumor stage, emergency surgery, duration of surgery, or amount of blood loss [1, 10–16]. Nevertheless, CAL rates remain stable over the past years [17]. AL after colon or colorectal resection is associated with a prolonged hospital stay, substantial negative impact on morbidity and mortality rates, as well as higher cancer recurrence frequency [1, 3, 18, 19]. Therefore, an objective of utmost importance is the early detection of AL to limit the negative postoperative outcome to a minimum. Occurrence of an anastomotic leakage is associated with a local inflammation at this site. Moreover, an upregulation of inflammatory cytokines and chemokines in case of inflammation is commonly known [20]. As acute-phase proteins, cytokines or chemokines, respectively, were synthesized in the liver and at the site of inflammation, protein levels taken from pelvic drain fluid represent the local milieu and could be more specific for the detection of an anastomotic leakage [21]. To investigate markers for AL, we analyzed a panel of inflammatory markers in sera and peritoneal fluid from the abdominal drain on the third postoperative day from patients with and without CAL after colorectal surgery due to colorectal cancer. CCL-1/I-309 (C-C motif ligand 1), CCL8/MCP-2 (monocyte chemotactic protein-2), CCL13/MCP-4 (monocyte chemotactic protein-4), CXCL5/ENA-78 (epithelial neutrophil-activating peptide), LIF (leukemia inhibitory factor), IL-16 (interleukin-16), and IL-21 (interleukin-21) were chosen for analysis. Selected markers were chosen based on a literature research because of their known role in inflammatory processes. CCL-1/I-309 is produced mostly by T_regs_ at the site of inflammation [22]. CCL8/MCP-2 activates immune cells like natural killer cells as a proinflammatory mediator [23]. CCL13/MCP-4 carries out proinflammatory actions through chemotaxis of monocyte-derived macrophages, lymphocytes, and basophils [24]. CXCL5/ENA-78 is detected in inflamed intestinal mucosa. Il-16 is produced by T lymphocytes, eosinophils, mast cells, and macrophages during inflammatory responses and is recruited if cell necrosis occurs [25, 26]. Il-21 expression is induced by other cytokines, e.g., Il-6, and it regulates the proliferation and function of numerous immune cells like natural killer cells [27]. And LIF promotes recruitment of inflammatory cells to the area of damage [28].Moreover, until now no study investigated the influence of these inflammatory markers in anastomotic leakage after colorectal surgery. We hypothesize that the above named inflammatory markers - measured in the peritoneal fluid - could predict the occurrence of an anastomotic leakage after colorectal surgery already on the third postoperative day, prior to the mean occurrence at the eight postoperative day.

## Material and methods

### Patients and samples

In total material of 92 patients with colorectal cancer who underwent surgery at the Department of General, Visceral and Transplantation Surgery, University of Heidelberg, Germany, in an elective setting was collected for this study. Patients were not gathered consecutively and not chronologically due to organizational reasons (e.g., if the third postoperative day fell on a weekend or public holiday). Therefore, it is not possible to deduce from the number of patients to the anastomotic leakage rate. Of these patients, 38 patients with a complete set of samples were selected and divided into two groups: patients without an anastomotic leakage (*n* = 20) and patients who developed an anastomotic leakage in the clinical course without other postoperative complications (*n* = 18). Anastomotic leakage was detected clinically, by endoscopic examination or CT scan with rectal contrast agent enema. Patients with secondary carcinomas, drainage removal before the third postoperative day, or postoperative complications other than anastomotic leakage like pneumonia, wound infection, or urinary tract infection were excluded in order to preferably recruit a homogenous patient collective. Potential bias of clinical parameters between AL and non-AL patients was analyzed for tumor localization, age, gender, BMI, smoker, TNM category, R, grading, and neoadjuvant therapy.

We hypothesize that other infections or inflammatory processes like surgical site infection, urinary tract infection, and pneumonias could falsify our measurement of the inflammatory markers with false higher results when there is an additional inflammatory process going on. Therefore, only patients with the postoperative complication of an anastomotic leakage were included without any other inflammatory morbidities in the postoperative course.

Peritoneal fluid was collected via routinely intraoperatively inserted abdominal drains on the third postoperative day prospectively. Patient’s sera were likewise collected on the third postoperative day. These samples were used for Multiplex ELISA and lab analysis. For the measurement of CRP (mg/dl), peritoneal fluid and serum samples of each patient were sent to the central laboratory, University of Heidelberg. Clinical and histopathological characteristics of the patients like age, gender, tumor location, TNM classification, UICC stage, R-classification, grading, neoadjuvant (radio) chemotherapy, and postoperative complications, especially anastomotic leakage, were obtained from a prospective clinical database for each patient. Every patient gave written informed consent and the local ethics committee approved the study (S-283/2012).

### Luminex®-based multiplex assay and lab analysis

Peritoneal fluid and serum samples for the detection of CCL-1/I-309 (C-C motif ligand 1), CCL8/MCP-2 (monocyte chemotactic protein-2), CCL13/MCP-4 (monocyte chemotactic protein-4), CXCL5/ENA-78 (epithelial neutrophil-activating peptide), LIF (leukemia inhibitory factor), IL-16 (interleukin-16), and IL-21 (interleukin-21) were processed using Milliplex MAP Human Cytokine/Chemokine Magnetic Panel II Assay Kit (Merck Millipore, Millipore Corporation, Billerica, MA, USA) according to the manufacturer’s protocol. The exact concentration of these markers (pg/ml) was detected in each sample by Luminex® 100™ reader (Luminex Corporation, Austin, TX, USA).

### Statistics

Statistical analyses were conducted with Excel 2013 (Microsoft Corporation, Redmond, WA, USA) and SPSS version 24 (SPSS, IBM Corporation, Armonk, NY, USA). Mann-Whitney *U*-test was used in a univariate analysis to determine expressional differences of CCL8/MCP-2, CXCL5/ENA-78, LIF, and CRP. Expressional data are presented as mean + SEM. Patients’ clinical characteristics regarding anastomotic leakage were calculated using Chi-Quadrat-Test. Area under receiver operating characteristic (ROC) curve analysis was conducted for evaluation of sensitivity and specificity of each marker. The cutoff value was determined by using Youden Index. Multivariate analysis was performed with linear regression model including CCL8/MCP-2, CXCL5/ENA-78, LIF, and CRP. Results were considered significant at a *p* value less than 0.05.

## Results

### Patients’ characteristics

**Fig. 1:**
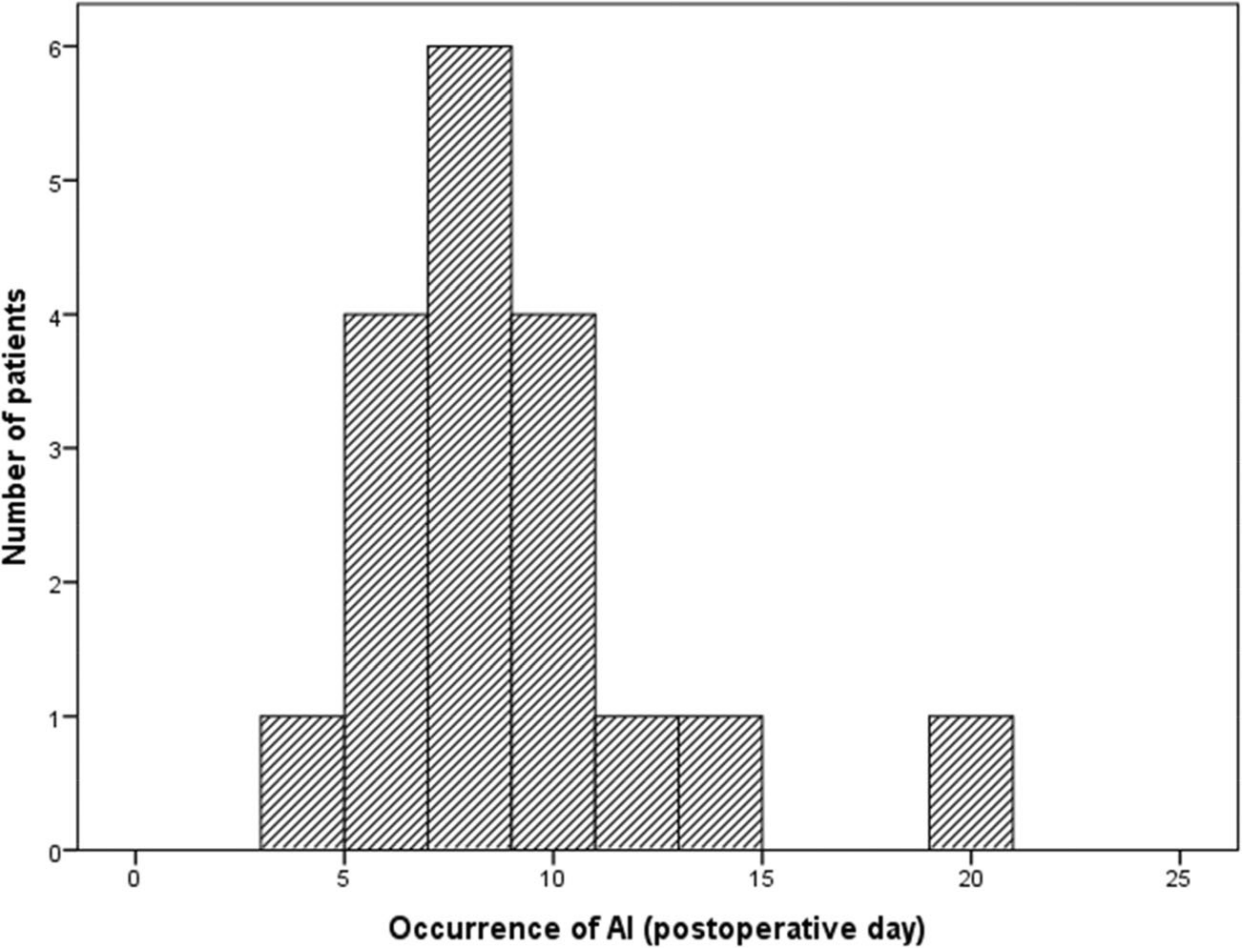
Histogram of the postoperative day of occurrence of the anastomotic leakage (*n* = 18). Median occurrence of an anastomotic leakage was on the eithth (IQR sixth–tenth) postoperative day

Thirty-eight patients who underwent surgery due to colon or colorectal adenocarcinoma were included into the study, 18 patients with and 20 patients without an anastomotic leakage. In cases of anastomotic leakage, median occurrence happened at the eighth (IQR sixth–tenth) postoperative day (Fig. 1). One patient revealed a Grade A, eight patients a Grade B, and nine patients a Grade C anastomotic leakage. Male patients (*p* = 0.046) and patients with a rectal anastomosis (*p* = 0.016) revealed a significantly increased risk of an anastomotic leakage, whereas there was no correlation between age (*p* = 0.492), BMI (*p* = 0.587); T (*p* = 0.253), N (*p* = 0.582), and M (*p* = 0.106) category; R status (*p* = 0.485); grading (*p* = 0.085); or receipt of neoadjuvant radio-/chemotherapy (*p* = 0.804) and the occurrence of an anastomotic leakage. Detailed patients’ characteristics are included in Table 1.

**Table 1:** Clinical and histopathological parameters of the patients and influence on anastomotic leakage

Patient characteristics	Number of patients (*n* = 38)	AL (*n* = 18)	Non-AL (*n* = 20)	*p* value
Tumor localization				
Colon	14 (36.8%)	3 (16.7%)	11 (55%)	0.014
Rectum	24 (63.2%)	15 (83.3%)	9 (45%)	
Median age				0.492
≤ 60 years	17 (44.7%)	7 (38.9%)	10 (50%)	
> 60 years	21 (55.3%)	11 (61.1%)	10 (50%)	
Gender				0.043
Male	28 (73.7%)	16 (88.9%)	12 (60%)	
Female	10 (26.3%)	2 (11.1%)	8 (40%)	
BMI [kg/m^2^]				0.587
< 25	16 (42.1%)	4 (22.2%)	6 (30%)	
≥ 25	22 (57.9%)	14 (77.8%)	14 (70%)	
Smoker				0.463
Yes	27 (71%)	6 (33.3%)	9 (45%)	
No	11 (29%)	12 (66.7%)	11 (55%)	
T category				0.253
T1	0 (0%)	0 (0%)	0 (0%)	
T2	7 (18.4%)	4 (22.2%)	3 (15%)	
T3	25 (65.8%)	13 (72.2%)	12 (60%)	
T4	6 (15.8%)	1 (5.6%)	5 (25%)	
N category				
N0	21 (55.2%)	9 (50%)	12 (60%)	0.582
N1	11 (29%)	5 (27.8%)	6 (30%)	
N2	6 (15.8%)	4 (22.2%)	2 (10%)	
M category				
M0	26 (68.4%)	10 (55.6%)	16 (80%)	
M1	12 (31.6%)	8 (44.4%)	4 (20%)	0.106
R				0.485
R0	35 (92.1%)	16 (88.9%)	19 (95%)	
R1	3 (7.9%)	2 (11.1%)	1 (5%)	
Grading				0.085
G1	0 (0%)	0 (0%)	0 (0%)	
G2	19 (50%)	7 (38.9%)	12 (60%)	
G3	5 (13.2%)	4 (22.2%)	1 (5%)	
n/a*	14 (36.8%)	7 (38.9%)	7 (35%)	
Neoadjuvant therapy				0.804
Yes	14 (36.8%)	7 (38.9%)	7 (35%)	
No	24 (63.2%)	11 (61.1%)	13 (65%)	

*n/a* not available; *due to neoadjuvant radiochemotherapy

### Expressional results of Luminex® 100™-based multiplex assay and lab analysis

**Fig. 2:**
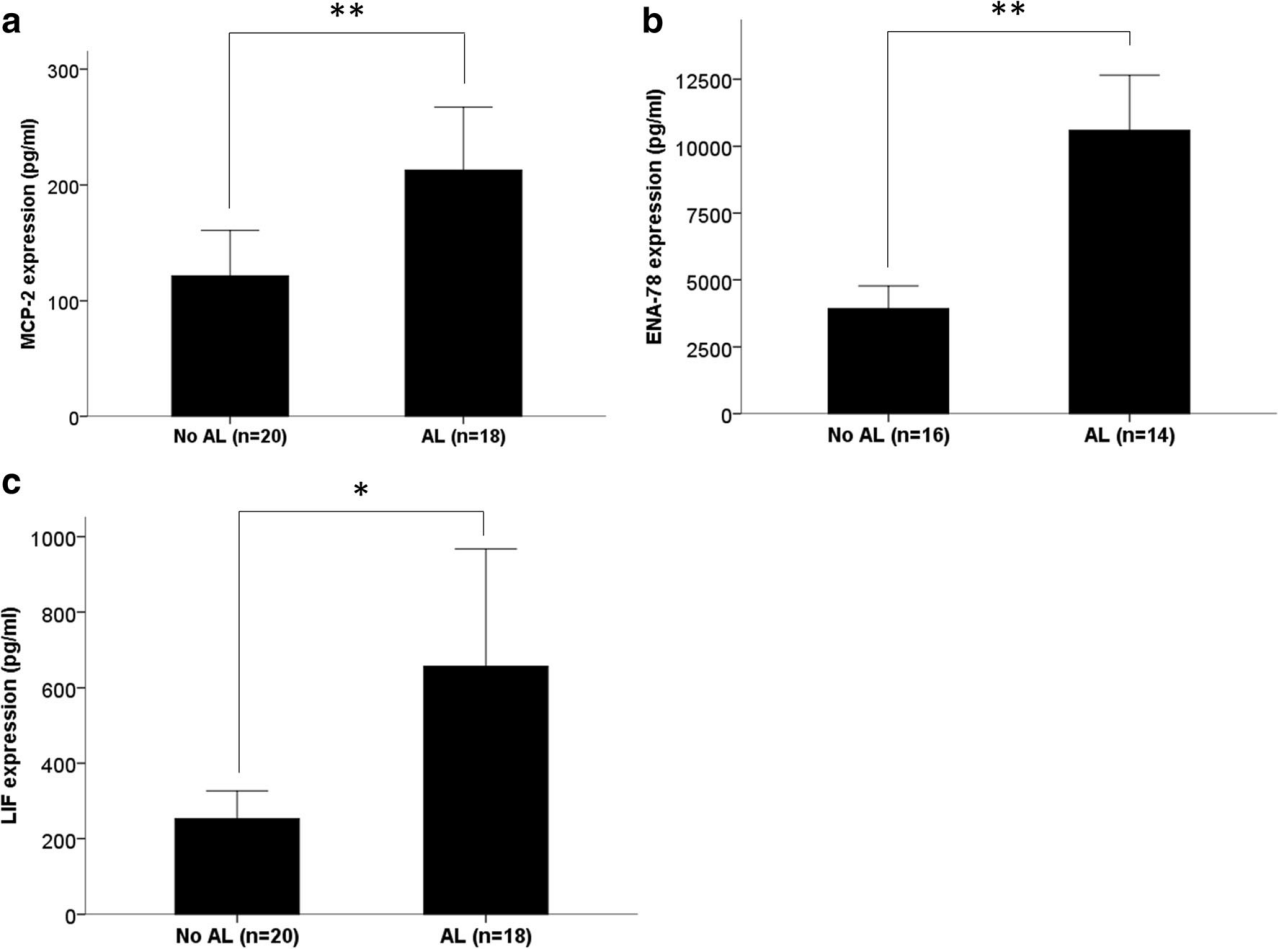
Expressional differences of CCL8/MCP-2, ENA-78, and LIF on the third postoperative day in peritoneal fluid samples of patients with and without an anastomotic leakage. CCL8/MCP-2, CXCL5/ENA-78, and LIF were significantly overexpressed in peritoneal fluid samples in patients developing an anastomotic leakage compared with patients without an anastomotic leakage. Bars represent mean + SEM

**Fig. 3:**
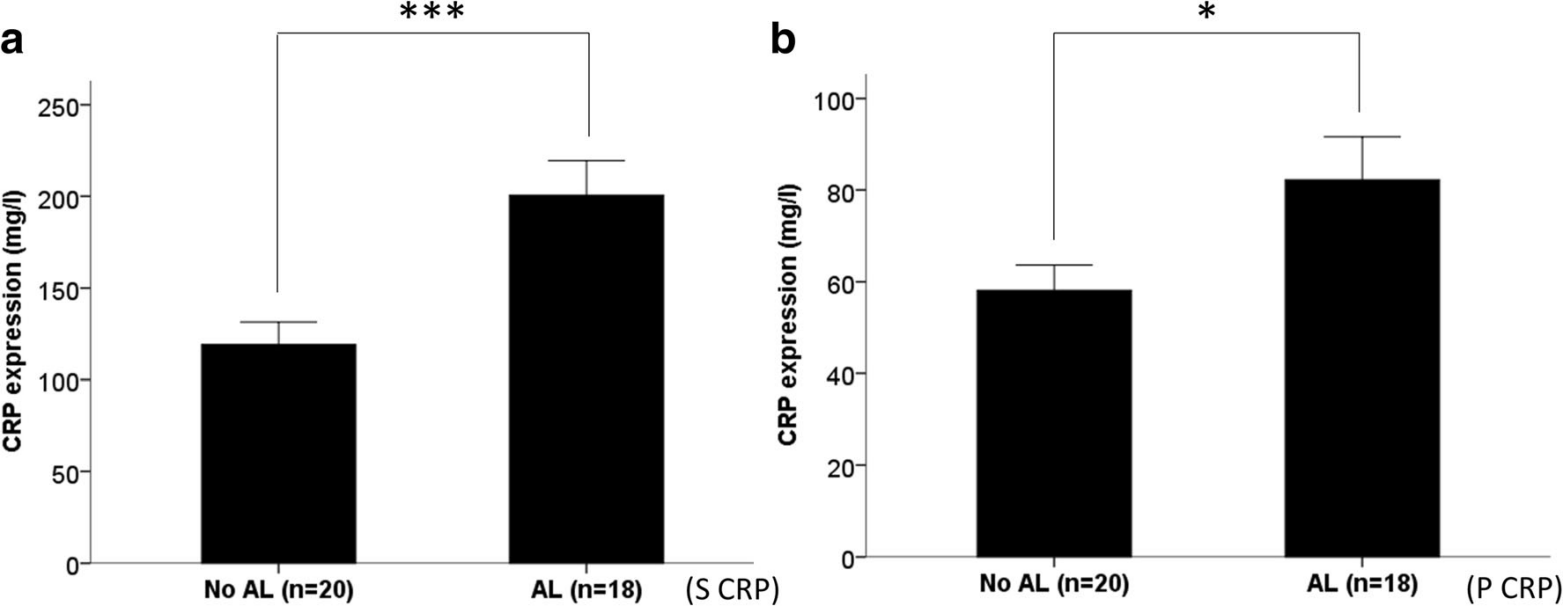
Expressional differences of CRP on the third postoperative day in peritoneal fluid and serum samples of patients with and without an anastomotic leakage. CRP was significantly overexpressed in peritoneal fluid and serum samples in patients developing an anastomotic leakage compared with patients without an anastomotic leakage. Bars represent mean + SEM

Luminex**®** 100™-based expression analysis revealed a significantly higher expression of CCL8/MCP-2 (*p* = 0.005) on the third postoperative day in peritoneal fluid samples of patients developing an anastomotic leakage (median CCL8/MCP-2 expression 115.28 pg/ml) compared with patients without an anastomotic leakage (median CCL8/MCP-2 expression 73.85 pg/ml). Also for CXCL5/ENA-78, significantly elevated levels were found on the third postoperative day in peritoneal fluid samples of patients developing an anastomotic leakage (median CXCL5/ENA-78 expression 9471.45 pg/ml) than those without (median CXCL5/ENA-78 expression 3601.66 pg/ml) (*p* = 0.005). Likewise, for LIF, we found a significantly higher expression in the peritoneal fluid of patients exhibiting an anastomotic leakage (median LIF expression 324.63 pg/ml) than those without an anastomotic leakage (median LIF expression 137.97 pg/ml) (*p* = 0.033) (Fig. 2). For CCL1/I-309 (*p* = 0.352), CCL-13/MCP-4 (*p* = 0.935), IL-16 (*p* = 0.534), and IL-21 (*p* = 0.206), no differential expression in peritoneal fluid samples of patients with and without anastomotic leakage was found. Moreover, lab analysis revealed significantly elevated CRP level in both peritoneal fluid (*p* = 0.033) and serum samples (*p* = 0.001) of patients with an anastomotic leakage compared with patients without an anastomotic leakage (median peritoneal fluid CRP expression 74.6 mg/dl in AL patients vs. median peritoneal fluid CRP expression in non-AL patients 53.6 mg/dl) (median serum CRP expression in AL patients 185.7 mg/dl vs. median serum CRP expression in non-AL patients 112.8 mg/dl) (Fig. 3). However, for none of the seven markers, a differential expression in serum samples of patients with and without anastomotic leakage was found: CCL8/MCP-2 (*p* = 0.478), CXCL5/ENA78 (*p* = 0.534), LIF (*p* = 1.0), CCL1/I-309 (*p* = 0.107), CCL-13/MCP-4 (p = 0.1), IL-16 (*p* = 0.747), and IL-21 (*p* = 0.363).

Taken together CCL8/MCP-2, CXCL5/ENA-78, LIF, and CRP were significantly elevated on the third postoperative day in the peritoneal fluid of patients who develop an anastomotic leakage compared with patients without an anastomotic leakage. For CRP this was shown in serum samples, too.

### Receiver operating characteristic analysis (ROC analysis)

**Fig. 4:**
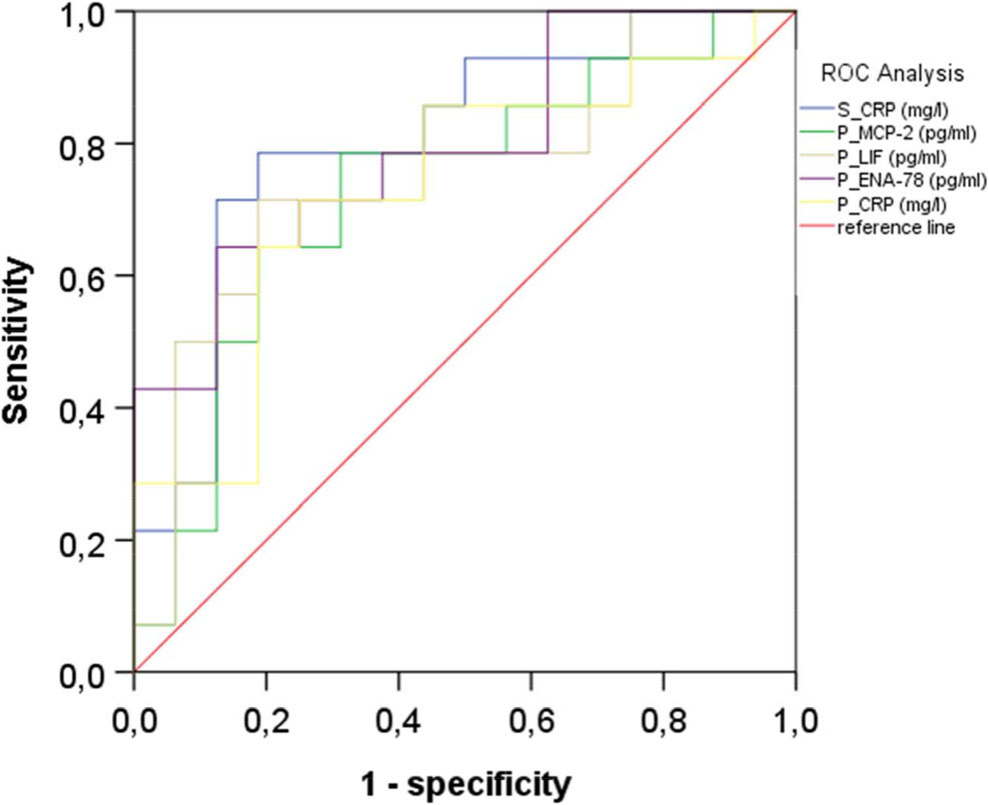
Area under the receiver operating characteristic curves (ROC analysis) for CCL8/MCP-2, CXCL5/ENA-78, and LIF in peritoneal fluid samples on the third postoperative day of patients developing an anastomotic leakage and patients without an anastomotic leakage; P, peritoneal fluid; S, serum

To evaluate the potential of the three identified peritoneal proteins as markers to differentiate between patients with and without an anastomotic leakage and to predict an anastomotic leakage on the third postoperative day, receiver operating characteristic analyses were performed. The assessment of sensitivity and specificity of CCL8/MCP-2, CXCL5/ENA-78, LIF, and CRP in peritoneal fluid samples and of CRP in serum samples is shown in Table 2 and Fig. 4. Moreover, we evaluated if the combination of peritoneal CCL-8/MCP-2, CXCL5/ENA-78, LIF, and peritoneal as well as serum CRP achieves a higher sensitivity and/or specificity than one marker alone. However, the combination of these markers revealed no better prediction (Table 2 and Supplementary Fig. 1). Subsequently, we performed a multivariate analysis of pf-CCL-8/MCP-2, pf-CXCL5/ENA-78, pf-LIF, pf-CRP, and s-CRP in order to evaluate if one of the tested markers could be an independent prognostic factor for the occurrence of an anastomotic leakage. But multivariate analysis failed to be significant (s-CRP, *p* = 0.211; pf-CRP, *p* = 0.521; CCL-8/MCP-2, *p* = 0.776; CXCL5/ENA-78, *p* = 0.134; LIF, *p* = 0.703).

**Table 2:** Receiver operating characteristic curve analysis of CCL8/MCP-2, CXCL5/ENA-78, and LIF in patients’ peritoneal fluid suffering anastomotic leakage

Biomarker	AUC	95% CI	Cutoff	Sensitivity (%)	Specificity (%)
Peritoneal fluid					
CCL8/MCP-2	0.764	0.607–0.921	89.99 pg/ml	83.3	70
CXCL5/ENA-78	0.795	0.633–0.956	6197.87 pg/ml	64.3	87.5
LIF	0.703	0.532–0.874	155.7 pg/ml	77.8	65
CRP	0.703	0.528–0.878	62.92 mg/l	72.2	75
Serum					
CRP	0.811	0.673–9.949	153 mg/l	77.8	80
Combined pf-CCL-8, pf-CXCL5, pf-LIF, pf-CRP, and s-CRP	0.786	0.62–0.951	2.9 × 10^−22^ mg^5^/ml^5^	85.7	68.7

*AUC* area under the curve, *cutoff* determined by using Youden Index, *pf* peritoneal fluid, *s* serum

## Discussion

The present study reveals altered expression of CCL8/MCP-2, CXCL5/ENA-78, and LIF in peritoneal fluid samples of patients with an anastomotic leakage after colorectal surgery.

Anastomotic leakage is still a detrimental complication after colorectal surgery with adverse prognostic effects for the patients including prolonged hospital stay as well as increased morbidity and mortality rates [1, 18]. Moreover, anastomotic leakage displays a risk factor for local recurrence and shortens long-term cancer-specific survival [29]. Clinical signs of an anastomotic leakage before the fifth postoperative day are unusual, and as mean day of diagnosis, the eighth postoperative day is stated commonly [8]. Notwithstanding the advancements in operative techniques and definition of risk factors, the incidence of anastomotic leakages after colorectal surgery has not dwindled over the last decade [1, 4, 30]. Additionally, a delay in diagnosis of an anastomotic leakage is common due to diagnostic methods lacking sensitivity and specificity [21, 31]. Doeksen et al. described a sensitivity of 54% and a specificity of 66% for the detection of a colorectal anastomotic leakage using CT scans. However, it is not described if a rectal filling with contrast agent was performed [32]. Hence, additional indicators for the detection of an anastomotic leakage are of utmost importance. A variety of studies evaluated several inflammatory markers like Il-6, TNF-α, MMP8, MMP9, or procalcitonin in peritoneal fluid and/or serum samples of patients suffering anastomotic leakage after colorectal resection in order to determine diagnostic biomarker [17, 33–38]. One of the most assessed markers constitutes CRP value. C-reactive protein is an acute-phase reactant protein which is synthesized mainly by hepatocytes but also by blood monocytes in response to infection or inflammation upon stimulation by proinflammatory cytokines as interleukin-6 (Il-6) and tumor necrosis factor-α (TNF-α) [39]. CRP activates complement pathway and boosts phagocytosis of damaged cells [40], while it is a reliable inflammatory marker, but non-specific [41]. Singh et al. defined in their systematic review and meta-analysis serum CRP on PODs (postoperative days) 3, 4, and 5 to be a diagnostic tool for the detection of an anastomotic leakage after colorectal surgery with an area under the curve of 0.81 and a CRP cutoff value of 172 mg/l on POD 3 [41]. Komen et al. assessed significantly elevated CRP level in peritoneal fluid samples on POD 3 and POD 5 in patients suffering an anastomotic leakage after colorectal surgery [9]. No area under the curve or cutoff values were defined in this study. Congruently with the abovementioned studies, we observed significantly elevated CRP levels on POD 3 in both serum and peritoneal fluid—with an area under the curve of 0.811 likewise and a CRP cutoff value of 153 mg/l and an area under the curve of 0.703 and a CRP cutoff value of 62.92 mg/l, respectively—of patients developing an anastomotic leakage compared with patients without an anastomotic leakage, indicating that our study collective constitutes a representative study cohort.

We found a differential expression of CCL8/MCP-2, CXCL5/ENA78, and LIF in peritoneal fluid samples of patients developing an anastomotic leakage on POD 3 compared with patients without an anastomotic leakage. As acute-phase proteins were synthesized in the liver and at the site of inflammation, protein levels taken from pelvic drain fluid represent the local milieu and could be more specific for the detection of an anastomotic leakage than serum markers [21]. Upregulation of cytokines and chemokines in case of inflammation is commonly known [20]. Induced by fibroblasts, macrophages, and endothelial cells, CCL8/MCP-2 acts through CCR1–3, CCR2, CCR3, and CCR5, activating immune cells including neutrophils, eosinophils, basophils, monocytes, T cells, and natural killer cells as a proinflammatory mediator [23, 42, 43]. Due to local inflammation at the site of the anastomotic leak, it is comprehensible that CCL8/MCP-2 is higher expressed in peritoneal fluid samples of patients developing anastomotic leakage. Moreover, the chemokine CXCL5/ENA-78 is higher expressed in patients with an anastomotic leakage, too. CXCL5/ENA-78 is an epithelial-derived chemokine appearing in the inflamed intestinal mucosa recruiting neutrophils from the lamina propria to the epithelial layer [44–46]. Further, it is produced by enterocytes, monocytes, and endothelial cells and constitutes a powerful neutrophil chemoattractant interacting with CXCR2 [44, 47–49]. And it drives through cancer-related angiogenesis [50]. LIF—a member if the IL-6 cytokine family—acts through LIF receptor ß which is located on macrophages and monocytes [51]. LIF activates STAT3/JAK1-kinase, PI3-kinase, and MAPK pathway and epitomizes proinflammatory characteristics like induction of acute-phase proteins and promotes recruitment of inflammatory cells to the area of damage [28, 52]. As for CCL8 and CXCL5, we found a higher expression of LIF in the peritoneal fluid samples of insufficient patients.

Taken together we found peritoneal fluid level of CCL-8/MCP-2, CXCL5/ENA-78, and LIF on the third postoperative day to be higher in patients with than without an anastomotic leakage. However, there are some limitations of the current study. First, the limited sample size of the small patient cohort due to prospectively collected probes could be an issue of confounding. Second, male sex, higher T status, and rectal anastomosis are known to be associated with higher AL rates and could depict confounder variables [1, 2, 4]. Therefore, we compared the clinical patient’s data with the anastomotic leakage rate. As described in the literature, in this study, male sex and rectal anastomosis were associated with an occurrence of an anastomotic leak. For the other tested clinical parameters like age, BMI, smoker, TNM category, R, grading, and neoadjuvant therapy, no association with the occurrence of an anastomotic leakage was assessed, resulting in a low risk of bias.

Third, only seven out of a high number of cytokines and chemokines were measured due to precise method of the Luminex® bead-based multiplex assay. Fourth, patients with other postoperative complications—like wound infection, urinary tract infection, pneumonia, and secondary carcinomas—were excluded in order to recruit a consistent homogenous patient collective with no other inflammatory postoperative comorbidities than AL. Hence, a difficulty could be to detect an anastomotic leakage with the assessed markers, if the patient has more than one postoperative complication. Therefore, further studies with a larger patient cohort are needed.

## Conclusions

This study demonstrates an elevated level of the inflammatory markers CCL8/MCP2, LIF, and CXCL5/ENA-78 on the third postoperative day in peritoneal fluid samples from patients developing anastomotic leakage after colorectal surgery compared with patients without anastomotic leakage. ROC analysis showed that CCL8/MCP2, LIF, and CXCL5/ENA-78 might be potential markers for an early detection of anastomotic leaks after colorectal surgery. Further studies with a larger patient cohort with inclusion of different variables are needed to evaluate their potential as predictive biomarkers for anastomotic leakage.

## Electronic supplementary material

ESM 1 (JPG 48 kb)
