# Supplementary figures and images for: Evaluation of the inflammatory markers CCL8, CXCL5, and LIF in patients with anastomotic leakage after colorectal cancer surgery
Source: Int J Colorectal Dis. 2020 Apr 19;35(7):1221–30. doi: 10.1007/s00384-020-03582-2 (PMC7320065; doi:10.1007/s00384-020-03582-2)

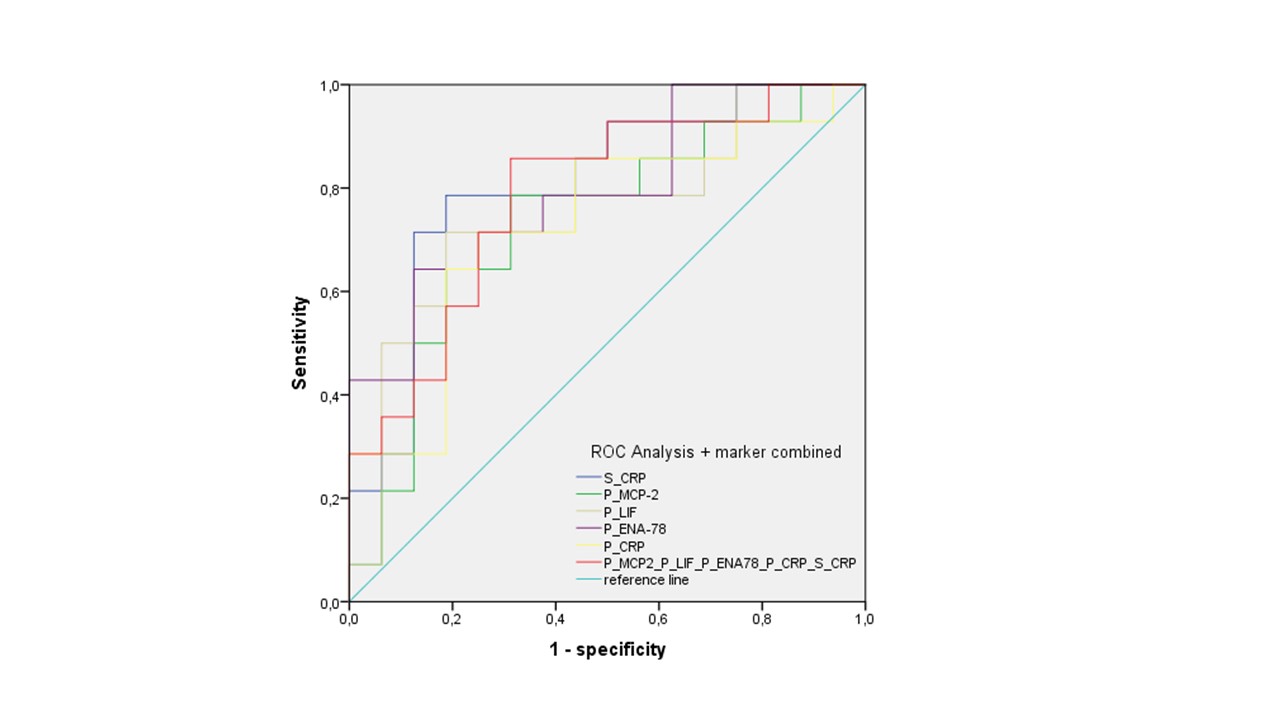

Supplement: Supplementary file 1 — (JPG 48 kb) [file 384_2020_3582_MOESM1_ESM.jpg]
